# Associations of *PI3KR1* and *mTOR* Polymorphisms with Esophageal Squamous Cell Carcinoma Risk and Gene-Environment Interactions in Eastern Chinese Populations

**DOI:** 10.1038/srep08250

**Published:** 2015-02-05

**Authors:** Jinhong Zhu, Mengyun Wang, Meiling Zhu, Jin He, Jiu-Cun Wang, Li Jin, Xiao-Feng Wang, Jia-Qing Xiang, Qingyi Wei

**Affiliations:** 1 Cancer Institute, Fudan University Shanghai Cancer Center, Shanghai, China; 2 Department of Oncology, Shanghai Medical College, Fudan University, Shanghai, China; 3 Molecular Epidemiology Laboratory and Laboratory Medicine, Harbin Medical University Cancer Hospital, Harbin, Heilongjiang, China; 4 Department of Oncology, Xinhua Hospital affiliated to Shanghai Jiaotong University, School of Medicine, Shanghai, China; 5 State Key Laboratory of Oncology in South China, Department of Experimental Research, Collaborative Innovation Center for Cancer Medicine, Sun Yat-Sen University Cancer Center, Guangzhou, Guangdong, China; 6 Department of Thoracic Surgery, Fudan University Shanghai Cancer Center, Fudan University, Shanghai, China; 7 Ministry of Education Key Laboratory of Contemporary Anthropology, State Key Laboratory of Genetic Engineering, School of Life Sciences, Fudan University, Shanghai, China; 8 Fudan-Taizhou Institute of Health Sciences, Taizhou, Jiangsu, China; 9 Duke Cancer Institute, Duke University Medical Center, Durham, NC, USA

## Abstract

Single nucleotide polymorphisms (SNPs) in the PI3K/PTEN/AKT/mTOR signaling pathway may contribute to carcinogenesis. We genotyped five potentially functional *PIK3R1* and *mTOR* SNPs in 1116 esophageal squamous cell cancer (ESCC) patients and 1117 cancer-free controls to assess their associations with ESCC risk. We observed no association with ESCC risk for any of the selected SNPs. However, the combined analysis of these SNPs revealed that subjects with one-to-three risk genotypes had an increased ESCC risk. Stratified analysis by body mass index (BMI) found that ESCC risk was significantly associated with each of three *mTOR* SNPs among subjects with BMI < 25.0. Specifically, we found that subjects carrying ≥ 1 risk genotypes had significantly increased ESCC risk, particularly for males, ever-smokers, ever-drinkers, and those with age > 60, or BMI < 25.0. Moreover, three *mTOR* haplotypes were associated with an increase in ESCC risk. Our meta-analysis of *mTOR* rs2295080 and cancer risk provided further evidence that *mTOR* SNPs might modulate cancer susceptibility. In this population, such risk effects might be modified by other risk factors, highlighting the importance of gene-environment interaction in esophageal carcinogenesis. Additional, larger studies are warranted to validate our findings.

Esophageal cancer is the sixth most common cause of cancer-related death in the world and also one of the most aggressive malignant tumors. According to GLOBOCAN 2008 estimates, approximately 482,300 new esophageal cancer cases and 406,800 deaths occurred in that year worldwide^1^. Esophageal cancer occurs as a result of multifaceted gene-environment interactions, as illustrated by its diverse patterns of incidence in different geographic regions of the world. The “esophageal cancer belt” (i.e., the highest risk area), including China, extends from Northern Iran through Central Asia to Northern-Central China. In particular, esophageal squamous cell carcinomas (ESCC) constitutes 90% of the cases in China^1^,^2^. Besides well-established risk factors, such as poor nutritional status, low intake of fruits and vegetables, smoking, alcohol intake, and drinking hot beverages, genetic variation in some key genes has been also suggested to modulate ESCC risk^3^,^4^. For example, numerous studies have demonstrated the existence of the association between ESCC risk and heritable genetic variants in genes involved in metabolism (e.g., *Glutathione S-transferase Mu 1 and Methylenetetrahydrofolate reductase*), DNA repair (e.g., *X-ray repair cross-complementing protein 1* and *Oxoguanine glycosylase*), and cell cycle control (*p53*)^3^,^4^,^5^. Apart from these, genetic variation in genes of other cancer-related pathways may also play a role in ESCC susceptibility.

Phosphatidylinositols (PtdIns) 3-kinases (PI3Ks), a family of lipid kinases, are divided into three different classes (I, II, and III), based on primary structure and biological features. Class I PI3Ks have been extensively studied because they are responsible for the production of PtdIns(3,4,5)P3 (also called PIP3)^6^. Class I PI3Ks are heterodimeric molecules comprising a regulatory subunit and a catalytic subunit. Among them, PI3K regulatory subunit 1 (alpha) (PIK3R1, alias: p85-α) and PI3K catalytic subunit alpha (PIK3CA, alias: p110-α) have been extensively studied, which are encoded by *PIK3R1* and *PIK3CA* genes, respectively^7^. PI3Ks debuted in the cancer research field back in the mid-1980s. Since then, the dysregulation of the PI3K/PTEN/AKT/mTOR pathway has been observed in a variety of human cancers, including cancers of the endometrium, stomach, lung, and esophagus^8^,^9^,^10^,^11^,^12^,^13^,^14^,^15^. Today, this pathway is well known to regulate important cellular events, including proliferation, adhesion, survival, and motility, which drive malignant transformation of cells and tumor progression^16^. Growth factors and hormones, such as epidermal growth factor receptor (EGFR) and insulin growth factor-1(IGF1), can stimulate class I PI3K by binding to the receptor tyrosine kinase (RTK). Activated class I PI3Ks convert PtdIns(4,5)P2 (called PIP2) to PIP3 by phosphorylating the hydroxyl group of the inositol ring of the former at the 3-position. The PIP3 then acts as a second messenger to trigger a downstream signaling cascade that is comprised of AKT, mTOR, and other proteins^16^. Mammalian target of rapamycin/FK506 binding protein 12-rapamycin associated protein 1 (mTOR/FRAP1), a serine/threonine kinase, is a member of the PI3Ks-related kinase protein family and is known as a central effector of cell growth and proliferation through the regulation of protein synthesis^17^.

Accumulating evidence has shown that mutations in some genes (*PIK3CA, RAS, PTEN*, and *AKT*) of the pathway could result in neoplastic transformation in both cellular and animal models^15^,^16^,^18^,^19^, suggesting a critical role of the pathway in carcinogenesis. Aberrant activation of this pathway has been closely related to various cancers, including ESCC. For example, it was reported that 11.5% of the tumors from ESCC patients harbored *PIK3CA* mutations^20^ and that the aberrant activation of mTOR occurred in 69.5% and 25% of ESCC in Japanese patients^11^ and Caucasian patients in the Netherlands^21^, respectively. The relatively high incidence of mutations in the PI3K pathway component provides strong evidence that dysregulation of this signaling pathway may contribute to the development of ESCC. Given the profound influence of aberrant activation of PI3K and mTOR on ESCC carcinogenesis, it is plausible that some potentially functional SNPs in genes encoding these proteins are likely to modulate ESCC susceptibility.

In contrast to extensive investigations regarding the mutations in these pathway genes, there are only a few studies exploring cancer risk associated with genetic variation in the same pathway genes. For example, Slattery et al. demonstrated that single nucleotide polymorphisms (SNPs) in *PIK3CA* and *mTOR/FRAP1* genes were significantly associated with risk of colon and rectal cancers, respectively^22^. Moreover, our group has previously reported associations of *mTOR* rs1883965 and rs2536 with risk of cancers of the esophagus, stomach, and prostate^9^,^23^,^24^. Two independent studies indicated that the *mTOR* rs1883965 SNP was significantly associated with an increased risk of gastric cancer^24^ and ESCC^23^.

In the present study, we expanded our previous studies by comprehensively analyzing additional potentially functional SNPs in the genes encoding class I PI3Ks and mTOR for their association with ESCC risk in an Eastern Chinese population.

## Results

### Characteristics of the Study Population

Overall, demographic characteristics of the case and cancer-free controls were comparable (Table 1). No statistically significant difference in the distributions of age and sex was observed between the cases and controls. With respect to drinking and smoking habits, more cases tended to be smokers and drinkers in comparison with controls. Moreover, a significantly higher percentage of cases had BMI (weight in kilograms/height^2^ in meters) below 25.0, when compared with the controls. To minimize a possible confounding effect, these variables were then adjusted for in the subsequent multivariate logistic regression analyses.

### Association between selected SNPs and ESCC risk

Three genes (*PIK3R1*, *PIK3CA*, and *mTOR*) were initially searched for potentially functional SNPs. However, we only investigated SNPs in the *PIK3R1* and *mTOR* genes in this study, because no potentially functional SNP in the *PIK3CA* gene met the SNP selection criteria. The genotype frequency distributions of all the selected SNPs in control subjects were in accordance with the Hardy Weinberg equilibrium (HWE). The minor allele frequencies (MAFs) of the SNP in these controls were similar to those reported in the CHB data from HapMap: 0.19 vs. 0.175 for rs3730089, 0.12 vs. 0.128 for rs3730090, 0.21 vs. 0.239 for rs2295080, 0.19 vs. 0.18 for rs1057079, and 0.078 vs. 0.109 for rs1064261, respectively.

Odds ratios (ORs) were determined by logistic regression analyses with adjustment for the covariates, i.e., age, sex, drinking status, smoking status, and BMI. Results including genotype frequencies, crude OR and 95% confidence interval (CI), and adjusted OR (95% CI) are shown in Table 2. Risk estimates indicated that none of the individual SNPs had a main effect on ESCC risk; that is, there was no significant association identified between any of the selected SNPs and ESCC risk. Next, SNPs under investigation were combined, and the risk estimates revealed that subjects with one, two, or three putative risk genotypes had significantly or borderline significantly increased risk of developing ESCC, compared with those without any such putative risk genotypes. Such an additive effect on risk did not seem to be risk-genotype dose-dependent, since there was no trend of increased ORs for individuals who carried one, two, or three risk genotypes (adjusted OR = 1.34, 95% CI = 1.07–1.68 for one risk genotype; adjusted OR = 1.42, 95% CI = 1.07–1.87 for two risk genotypes; adjusted OR = 1.29, 95% CI = 0.99–1.67 for three risk genotypes; Table 2). Furthermore, all participants were dichotomized according to the presence of risk genotypes. Subjects in the reference group had no risk genotypes, while subjects having one or more risk genotypes were categorized into the other group. Compared with the reference group, those with one or more risk genotypes had statistically, significantly increased ESCC risk (adjusted OR = 1.33, 95% CI = 1.10–1.62).

### Stratified Analysis

In an attempt to further scrutinize potential associations between the selected SNPs and ESCC risk, the data were stratified by the dichotomized variables of age, sex, smoking status, drinking status, and BMI, individually, under the dominant genetic model. No significant ESCC risk associated with any of *PIK3R1* and *mTOR* SNPs was detected in the dichotomized subgroups by age, sex, smoking, and drinking status (Tables 3,4,5). However, significant ESCC risk associated with three *mTOR* SNPs, but not *PIK3R1* SNPs, was each individually observed among subjects with BMI < 25.0 under the dominant genetic model (WV/VV vs. WW) (rs2295080: adjusted OR = 1.36, 95% CI = 1.07–1.73; rs1057079: OR = 1.31, 95% CI = 1.03–1.67, rs1014261: OR = 1.39, 95% CI = 1.01–1.92) (Tables 3,4,5).

Since the three *mTOR* SNPs were not in complete LD, their individual effects might be additive. We further explored the combined effects of these three SNPs in stratified analyses by age, sex, smoking status, drinking status, and BMI and found that significantly increased ESCC risk was identified for subjects carrying at least one of the three putative risk genotypes (i.e., rs2295080 GT/GG, rs1057079 CT/CC, and rs1014261 AG/GG) among the following subgroups: >60 years of age (adjusted OR = 1.28, 95% CI = 1.01–1.65), males (adjusted OR = 1.28, 95% CI = 1.05–1.56), ever-smokers (adjusted OR = 1.31, 95% CI = 1.03–1.67), ever-drinkers (adjusted OR = 1.34, 95% CI = 1.00–1.79) or BMI < 25.0 (adjusted OR = 1.52, 95% CI = 1.20–1.94) (Table 5). Moreover, while evaluating the strength of associations between *mTOR* SNPs and ESCC risk among subgroups with BMI < 25.0, the OR (1.52, 95% CI = 1.20–1.94) of combined risk genotypes was larger than the ORs (1.31, 95% CI = 1.03–1.67 for rs1057079; 1.39, 95% CI = 1.01–1.92 for rs1014261; 1.36, 95% CI = 1.07–1.73 for rs2295080) of any individual risk genotype (Table 5), indicating that there was likely a combined effect of these three SNPs. These findings suggested that the effect of each SNP is likely necessary but not sufficient, depending on the presence of other genetic variants.

### Association of High-Order Interactions with ESCC Risk by Multiple Dimension Reduction (MDR) Analysis

To further investigate the existence of possible gene-environmental interaction in association with ESCC risk, high-order interactions assessed by using the MDR analysis was performed with inclusion of the five selected SNPs (i.e., rs3730089, rs3730090, rs2295080, rs1057079, and rs1014261) and five known risk factors (i.e., age, sex, smoking status, drinking status, and BMI). In the MDR analysis, BMI was the best one-factor model with the highest cross-validation consistency (CVC) and the lowest prediction error among all ten factors, indicating that BMI was the strongest risk factor for ESCC. Moreover, the ten-factor model had a maximum CVC and a minimum prediction error, with the prediction error being statistically significant (Table 6). Taken together, the ten-factor model showed a better prediction than the one-factor model and represented the best model to predict ESCC risk for this study population.

### *mTOR* Haplotypes and ESCC Risk

Since *PIK3R1* and *mTOR* are located in different chromosomes, we only explored whether the haplotypes of three *mTOR* SNPs would influence ESCC risk. As presented in Table 7, seven *mTOR* haplotypes were identified. When the most frequent haplotype T-T-A was used as the reference group, three haplotypes, T-C-A, T-C-G, and G-T-A, were significantly associated with increased ESCC risk.

### Gene-Gene and Gene-Environment Interactions

As presented in Table 8, logistic regression analyses identified significant gene-environment interactions of BMI with either *mTOR* rs1057079 or rs2295080 SNPs. Moreover, our results further showed significant gene-gene interactions of *mTOR* rs2295080 with either *mTOR* rs1057079 or rs1064261. Interestingly, the interaction between smoking status and drinking status were also noticeable (Table 8).

### Meta-analysis for the Association between *mTOR* rs2295080 and Cancer Risk

**Figure 1:**
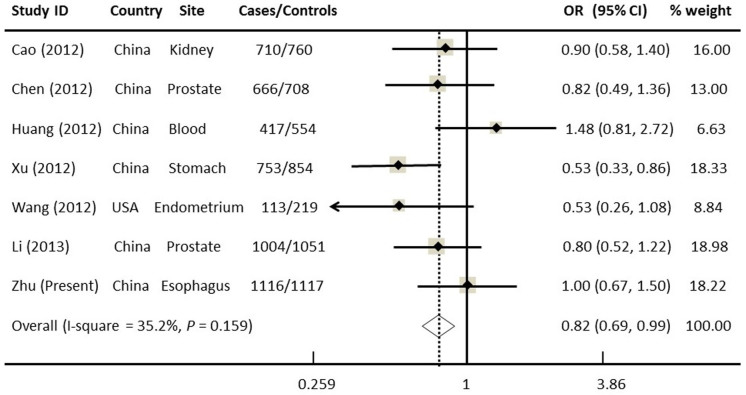
Forest plot of overall cancer risk associated with *mTOR* rs2295080 (a genetic recessive model). This was derived from a meta-analysis of seven relevant case-control studies. The OR and 95% CI of each study are plotted with a box and a horizontal line. Quadrangles represent pooled ORs and 95% CIs; Chi^2^, chi-square; df, degrees of freedom; I^2^, index of heterogeneity.

To date, six published studies have explored the association of *mTOR* rs2295080 with the risk of various cancers but yielded conflicting results^8^,^9^,^10^,^13^,^25^,^26^, whereas fewer studies on other *mTOR* SNPs have been published. To better evaluate such an association, we performed a meta-analysis with all published studies and our new data, leading to a total of 4772 cases and 5264 controls. When all the data were combined, the *mTOR* rs2295080 SNP appeared to be modestly protective and significantly associated with a decreased cancer risk under most of the genetic models tested without obvious among-study heterogeneity (homozygous: OR = 0.79, 95% CI = 0.66–0.95; heterozygous: OR = 0.88, 95% CI = 0.78–1.02, dominant: OR = 0.87, 95% CI = 0.80–0.94, recessive: OR = 0.82, 95% CI = 0.69–0.90) (Figure 1). Moreover, the shape of funnel plots seemed symmetrical and Egger's test showed no significance (data not shown), suggesting no publication bias.

## Discussion

Knowledge on the genetics of cancer can help health care professionals in providing high-risk individuals with better decisions on prevention and intervention strategies, such as cancer screening, early detection, and targeted therapy. Numerous studies have indicated that potentially functional SNPs in the important genes may confer host genetic susceptibility to cancer. Aberrant activation of the PI3K/PTEN/AKT/mTOR signaling pathway is a common event in a wide range of tumor types, suggesting a role for this pathway in carcinogenesis. In the present study, however, none of the studied SNPs in the *PIKR1* and *mTOR* genes exhibited an association with ESCC risk. However, the combined analysis of these SNPs revealed significant risk associations with one, two, and three risk genotypes, compared with zero risk genotype. Actually, lack of the main effect of individual SNPs on cancer risk does not necessarily rule out these SNPs as etiologic factors, because these SNPs may have low penetrance in cancer susceptibility, compared with environmental and life style factors contributing to the risk. It is likely, however, that the relevant exposure, such as smoking, alcohol intake, and hormonal disorder, may interact with genetic factors^27^. This was the case in the present study. For example, our stratified analysis by BMI suggested a significantly increased ESCC risk associated with *mTOR* rs2295080, rs1057079, and rs1014261, individually, among subjects with BMI<25.0. When risk genotypes of these three *mTOR* SNPs were combined, subjects with ≥1 risk genotype exhibited an increased ESCC risk in the older participants (age>60), males, smokers, drinkers, or those with BMI<25.0, supporting gene-environment interactions on ESCC susceptibility.

Although few studies have investigated cancer risk associated with the SNPs we studied, some reported findings that are in line with ours. For example, one study observed an association between *mTOR* rs2295080 and a reduced risk of renal cancer in 710 cases and 760 controls^25^. In addition, *mTOR* rs2295080 was also shown to protect against gastric cancer risk in a Chinese population^10^. Functional analysis demonstrated that the rs2295080 variant G allele reduced transcriptional activity in both normal gastric mucosa epithelial cell lines (GES-1) and three different gastric cancer cell lines, compared with the wide-type T allele^10^. Moreover, *mTOR* mRNA expression levels in gastric cancer tissues with GT/GG genotypes were significantly lower than those with the TT genotype^10^, indicating that *mTOR* rs2295080 may decrease gastric cancer risk by affecting *mTOR* transcription.

Similarly, our meta-analysis of seven studies with 4772 cases and 5264 controls found that rs2295080 was significantly associated with a reduced cancer risk under homogenous (GG vs.TT) and recessive (GG vs.TT/TG) genetic models. However, due to the relatively small sample size in the current meta-analysis, large single studies with different cancer types and ethnic groups are needed to validate our findings. Moreover, additional meta-analyses with stratified analyses by cancer type are warranted to further determine the effect of this SNP on the risk of each specific cancer. For example, in contrast with other cancers, our study indicated that *mTOR* rs2295080 variant genotypes (GT/GG) were associated with an increased ESCC risk, and the association became significant among subjects with BMI<25.0. One explanation for the discrepancy in the association studies is that the function of rs2295080 SNP may be tissue-specific, but the biological function of this SNP should be further examined in different types of cancer in future studies. It was not possible to perform additional meta-analyses for the remaining studied SNPs, because very few published studies (<3) have investigated the associations between each of those SNPs and cancer risk.

One earlier U.S. study of 1574 colon cancer cases and 1940 healthy controls revealed a significant association between *mTOR* rs1057079 variant genotypes and increased risk of colon and rectal cancers, while this SNP was also associated with tumor harboring *TP53* mutations^22^. The same study also found an association of *PIK3CA* rs7651265 SNP with rectal cancer risk^22^. However, in the present study, significant associations were only confined to certain subgroups exposed to smoking or alcohol, indicating the importance of considering other risk factors when analyzing genetic impact on cancer predisposition. As mentioned above, age and sex also appeared to modify the effect of genetic variants on ESCC risk. Although mechanisms remain unclear, ESCC is far more prevalent among males than females globally^1^. For example, a large-scale epidemiological study in North China observed that ESCC was more prevalent in males than females and in older populations rather than younger populations^28^. In the case that *mTOR* SNPs of interest only have some moderate effects, it is not unreasonable that the associations between the combination of these SNPs and ESCC risk were only detected in males and older groups who may have been exposed to environmental risk factors for a longer period. Therefore, it is plausible that our stratified analyses revealed an association between a combination of three *mTOR* SNPs and an increased ESCC risk among smokers and drinkers, since smoking and alcohol drinking are well-recognized strong risk factors for ESCC^29^. We also found that there was significant interaction between smoking and drinking in this study population. These finding were consistent with those reported by others, in which a meta-analysis suggested that tobacco and alcohol consumption synergistically increased the risk of developing ESCC^30^.

In the present study, significant *mTOR* SNP-related increases in ERCC risk were detected among subjects with BMI<25, but not among those with BMI ≥ 25.0, suggesting that BMI was a significant effect modifier of ERCC risk associated with *mTOR* SNPs. To support this finding, further MDR testing of the high-order interaction analysis consistently recognized BMI as a main risk factor for ESCC, which is consistent with the fact that body weight is reversely associated with risk of ESCC as well as with smoking, drinking, and nutrition. For example, previous studies conducted in both Western countries^31^,^32^ and China found that both poor nutrition and low BMI were associated with an increased ESCC risk. In the present study, we found a significant interaction between BMI and either *mTOR* rs1057079 or rs2295080 SNPs. Our results also showed that *mTOR* rs2295080 significantly interacted with either *mTOR* rs1057079 or rs1064261, suggesting that these SNPs of interest might collectively confer and modulate ESCC susceptibility.

There are some limitations in the present study. First, although age, sex, smoking, drinking, and BMI were considered, there were also a number of uncollected factors contributing to ESCC risk, including nutrition status; intake of hot beverages, fruits, vegetables; other genetic variations; and socioeconomic status. Failure to adequately control for these factors limited our ability to analyze gene-gene and gene-environment interactions. Second, the number of SNPs genotyped in the manuscript was also very limited, and some potentially functional SNPs in these two genes might be missed. Third, we performed multiple comparisons (single SNPs, SNPs combined, haplotypes, stratified by age, sex, BMI, etc.) in the present study, which may have led to chance findings (e.g., false positive findings). Therefore, these results should be interpreted with caution. Larger, more stringently designed studies are needed to validate our findings. Moreover, *PIK3CA* is a well-known oncogene, the activation of which has been implicated in various cancers. Failure to investigate the association of *PIK3C*A polymorphisms with ESCC risk is also a potential limitation.

In summary, we found that rs2295080, rs1057079, and rs1064261 SNPs in the *mTOR* gene may modify the host's genetic susceptibility to ESCC risk; however, these effects were largely dependent on other risk factors, i.e., BMI, age, sex, smoking and drinking status. Our results emphasize the importance of gene-environment interactions in determining the ESCC susceptibility, supporting the idea that the low-penetrant genetic effects of common SNPs on cancer predisposition may be fundamentally governed by the interplaying of SNPs and specific environmental exposures during the process of carcinogenesis.

## Methods

### Study Population

This case-control study was conducted at Fudan University, Shanghai Cancer Center. Briefly, the cases (*n* = 1116) were patients with newly-diagnosed and histopathologically confirmed ESCC from March 2009 to September 2011, who were all genetically unrelated Han Chinese and residents in Eastern China. The patients who had one or more of the following features were excluded: other types of cancer, primary tumors outside the esophagus, and cancers with unknown primary sites. Age and sex-matched, cancer-free controls (n = 1117) were selected from the Taizhou cohort^33^ following a procedure of matching with cases on age (± 5 years) and sex. A structured questionnaire was used to obtain the following information from each of the participants during personal interviews: demographic data and environmental exposure history such as age, sex, ethnicity, BMI, and tobacco and alcohol consumption. We defined individuals who smoked <100 cigarettes in their lifetime as “non-smokers”, while others were considered “smokers”. Moreover, subjects with alcohol consumption at least once a week for ≥1 year were defined as “drinkers”, and others were “non-drinkers”^34^. BMI was calculated from self-reported height and weight. We used a BMI value of 25 to divide subjects into two groups with BMI <25 and ≥25^34^; the World Health Organization (WHO) recommends body mass index (BMI, in kg/m^2^) ≥25 as a cutoff for categorization of overweightness^35^. Ninety percent of interviewed subjects consented to participate in this study by signing a written informed consent form. This research protocol was approved by the Institutional Review Board of FUSCC and the experiment on humans was performed in accordance with relevant guidelines and regulations.

### SNP Selection and Genotyping

**Figure 2:**
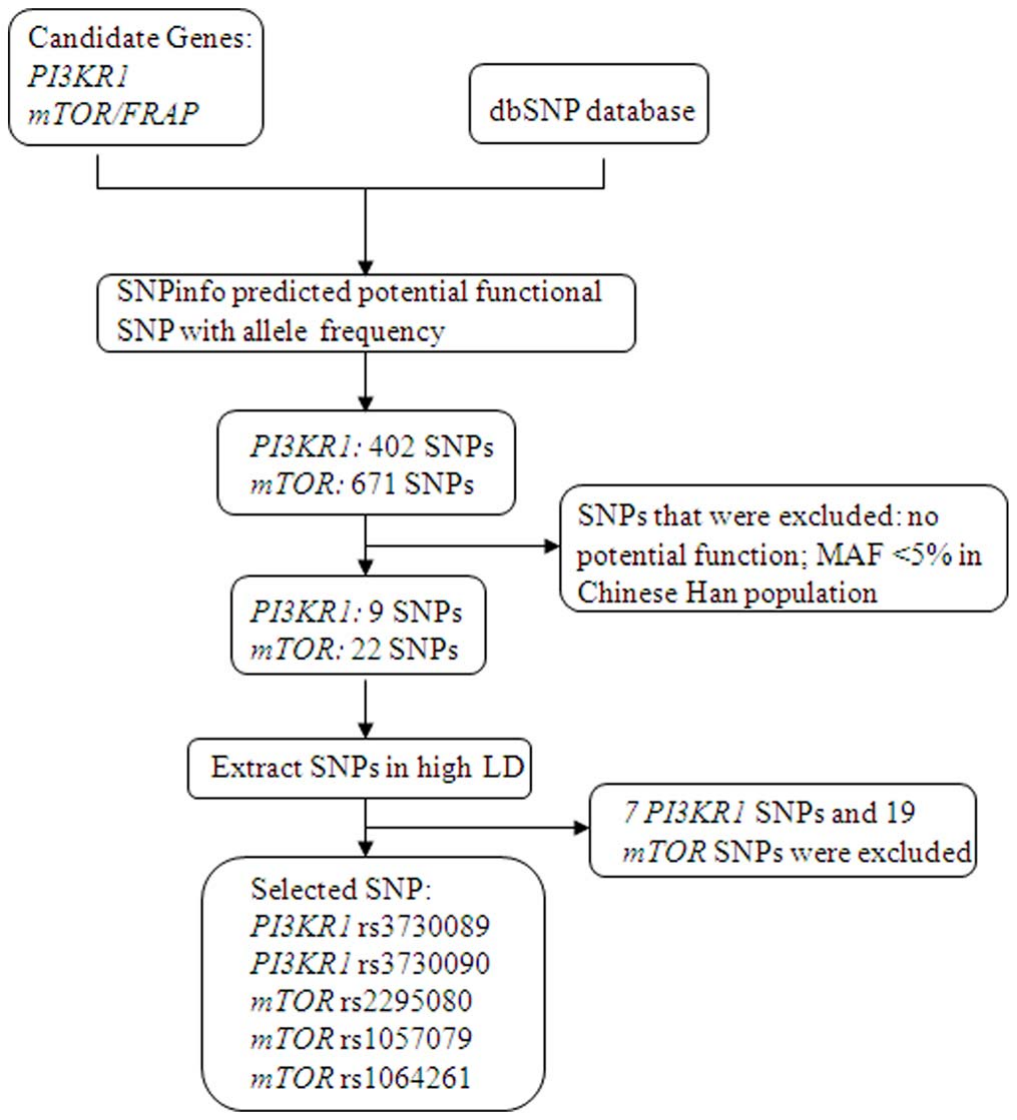
The SNP selection flow chart.

We searched the National Center for Biotechnology Information dbSNP database (http://www.ncbi.nlm.nih.gov/projects/SNP) for common, potentially functional SNPs in the *PIK3CA, PIK3R1 and mTOR* genes based on the following criteria: (1) located at exons, the 5′ near gene, 5′ untranslated regions (UTR), 3′ UTR, 3′ near gene and splice sites; (2) the minor allele frequency (MAF) ≥ 5% in Chinese Han population; (3) potentially functional SNPs as predicted by SNPinfo software (http://snpinfo.niehs.nih.gov/snpinfo/snpfunc.htm); (4) having low linkage disequilibrium (LD) with each other using an r^2^ threshold of <0.8; and (5) not investigated in the published genome-wide association studies (GWAS) of ESCC. No SNP in the *PIK3CA* gene met the criteria. Ultimately, we chose five SNPs (*PIK3R1*: rs3730089 and rs3730090; *mTOR*: rs1057079, rs1064261, and rs2295080) for the study. The SNP selection process is indicated in Figure 2.

We isolated genomic DNA from blood samples by using the Qiagen Blood DNA Mini Kit (Qiagen Inc., Valencia, CA) and performed the TaqMan assay for genotyping as reported previously^36^. Briefly, we labeled allele-specific probes for SNPs of interest with the fluorescent dyes VIC and FAM. During extension, the 5′- exonuclease activity of the Taq polymerase cleaves the fluorophore from the nonfluorescent quencher. By using the ABI 7900 HT Sequence Detection System (Applied Biosystems, Foster City, CA), we used a post-amplification allelic discrimination run on the machine to determine genotype based on the relative amount of fluorescence of VIC and FAM. Finally, we performed PCR reactions in a total reaction volume of 5 μl in 384-well plates. Individuals involved in genotyping were blind to patient status.

### Statistical Methods

We used the χ^2^ test to assess differences in the frequency distributions of the selected demographic variables, risk factors, and genotypes of the selected SNPs between the cases and controls. We tested the Hardy–Weinberg equilibrium (HWE) for genotype distribution in controls by a goodness-of-fit χ^2^ test. Crude and adjusted ORs and their 95% CIs for the association of ESCC risk with selected SNPs were calculated using both univariate and multivariate logistic regression analyses with adjustment for co-variates including age, sex, smoking, drinking, and BMI, respectively. These co-variates were selected because of their importance in possible interaction with genetic factors, and were entered into the model at the same time as a group of categorical variables defined in Table 3. The *P*-value for multiplicative interaction between these selected SNPs and co-variates (age, sex, BMI, etc) was calculated by adding the product terms to the logistic regression model. A two-tailed *P* < 0.05 was used as the criterion of statistical significance. We also evaluated the associations in stratified analyses by age, sex, BMI, and smoking and drinking status. Four genetic models, 1) homozygous (WW vs. VV), 2) heterozygous (WW vs. WV), 3) dominant (WW vs. WV/VV), and 4) recessive (WW/WV vs. VV), were adopted for these analyses, with W and V representing wild and variant alleles of each SNP, respectively. In the present study, we defined the haplotype as a combination of rs2295080, rs1014261, and rs1057079 SNPs in the *mTOR* gene. The unphased genotype data were used to determine haplotype frequencies and individual haplotypes. Logistic regression analysis was performed to calculate ORs for the association of haplotypes with ESCC risk, while the haplotype of the highest frequency was considered as the reference group. Moreover, genotypes with one or two variant alleles of a SNP were referred to as risk genotype. Risk genotypes used for the calculation were *PIK3R1* rs3730089 AG/AA, *PIK3R1* rs3730090 CT/TT, *mTOR* rs2295080 GT/GG, *mTOR* rs1057079 CT/CC, *mTOR* rs1064261 AG/GG. All tests were two-sided, and a *P*< 0.05 was considered statistically significant. All statistical analyses were performed with SAS software (version 9.1; SAS Institute, Cary, NC). Furthermore, we used MDR software (V2.0 beta 8.2) to determine the possible high-order gene-gene or gene-environment interactions in the association^37^. Briefly, we tested 100-fold cross-validation and 1000-fold permutation under the assumption of no association. The best candidate interaction model was supposed to have the minimum average prediction error and the maximum CVC.

Finally, a meta-analysis was conducted to evaluate the association between *mTOR* rs2295080 SNP and cancer risk. Briefly, the search terms, inclusion, and exclusion criteria were defined in previous publications^38^,^39^. Relevant studies were searched from the MEDLINE, EMBASE and Scopus databases (Last Updated: July 25, 2014). The pooled ORs and 95% CIs were calculated under four different genetic models. Chi-square-based Q-test was used to check heterogeneity assumption. Either the fixed-effects model (the Mantel-Haenszel method) or the random-effects model (the DerSimonian and Laird method) was applied to calculate the pooled OR estimate, depending on the heterogeneity of study populations included in this meta-analysis^36^,^37^. Potential publication bias was estimated by the funnel plot and Egger's linear regression test. Sensitivity analysis was performed to assess the stability of the meta-analysis. All statistical tests were performed with STATA (version 11.0; Stata Corporation, College Station, TX). All *P* values were two-sided, and a *P*<0.05 was considered statistically significant.

## Author Contributions

Conceived and designed the experiments: Q.Y.W., J.Q.X. Performed the experiments: J.H.Z., M.Y.W. Analyzed the data: J.H.Z., M.Y.W. Contributed reagents/materials/analysis tools: M.L.Z., J.H., J.C.W., J.L., X.F.W., Q.Y.W. Wrote the paper: J.H.Z., M.Y.W., J.Q.X., Q.Y.W.

## Figures and Tables

**Table 1:** Frequency distributions of selected characteristics of ESCC cases and controls

Variables	Cases No. (%)	Controls No. (%)	*P^a^*
All subjects	1,116 (100.0)	1,117 (100.0)	
Age, yr			0.890
Range	37–88	32–86	
Mean^b^	60.4 ± 8.3	60.3 ± 10.2	
Age group			
≤50	143 (12.8)	151 (13.5)	
51–60	423 (37.9)	405 (36.3)	
61–70	411 (36.8)	405 (36.3)	
>70	139 (12.5)	156 (13.9)	
Sex			0.410
Males	897 (80.4)	882 (79.0)	
Females	219 (19.6)	235 (21.0)	
Drinking status			<0.0001
Ever	498 (44.6)	369 (33.0)	
Never	618 (55.4)	748 (67.0)	
Smoking status			0.0038
Ever	681 (61.0)	614 (55.0)	
Never	435 (39.0)	503 (45.0)	
Pack-years			<0.0001
0	435 (39.0)	503 (45.0)	
≤16 (mean)	150 (13.4)	246 (22.0)	
>16 (mean)	531 (47.6)	368 (33.0)	
Body mass index			<0.0001
<25.0	721 (64.6)	485 (43.4)	
≥25.0	395 (35.4)	632 (56.6)	

^a^Two-sided *χ^2^* test for distributions between cases and controls.

^b^Data were presented as mean ± SD.

**Table 2:** Logistic regression analysis of associations between the genotypes of *PIK3R1* & *mTOR* and ESCC risk

Variants	Genotypes	Cases (N = 1,116)	Controls (N = 1,117)	*P*^a^	Crude OR (95% CI)	*P*	Adjusted OR (95% CI)	*P*^b^
*PIK3R1* rs3730089								
	GG	736 (66.0)	729 (65.4)	0.880	1.00		1.00	0.763
	AG	331 (29.7)	345 (30.9)		0.95 (0.79–1.14)	0.594	0.98 (0.81–1.19)	0.857
	AA	48 (4.3)	41 (3.7)		1.16 (0.76–1.78)	0.496	1.19 (0.76–1.85)	0.452
	AG/AA	379 (34.0)	386 (34.6)		0.97 (0.82–1.16)	0.755	1.00 (0.84–1.20)	0.972
*PIK3R1* rs3730090								
	CC	837 (75.3)	849 (76.4)	0.463	1.00		1.00	0.475
	CT	255 (23.0)	249 (22.3)		1.04 (0.86–1.27)	0.708	1.04 (0.85–1.28)	0.690
	TT	19 (1.7)	14 (1.2)		1.38 (0.69–2.76)	0.369	1.34 (0.65–2.75)	0.434
	CT/TT	274 (24.7)	263 (23.6)		1.06 (0.87–1.28)	0.578	1.06 (0.87–1.30)	0.570
*mTOR* rs2295080								
	TT	674 (60.6)	702 (63.1)	0.304	1.00		1.00	0.202
	GT	390 (35)	362 (32.5)		1.12 (0.94–1.34)	0.199	1.13 (0.94–1.36)	0.185
	GG	49 (4.4)	49 (4.4)		1.04 (0.69–1.57)	0.841	1.12 (0.73–1.71)	0.614
	GT/GG	439 (39.4)	411 (36.9)		1.11 (0.94–1.32)	0.222	1.13 (0.95–1.36)	0.167
*mTOR* rs1057079								
	TT	702 (63.0)	725 (65.0)	0.321	1.00		1.00	0.248
	CT	367 (32.9)	349(31.3)		1.09 (0.91–1.30)	0.368	1.10 (0.91–1.32)	0.322
	CC	45 (4.1)	41 (3.7)		1.13 (0.73–1.75)	0.573	1.18 (0.75–1.85)	0.484
	CT/CC	412 (37.0)	390 (35.0)		1.09 (0.92–1.30)	0.324	1.11 (0.93–1.33)	0.261
*mTOR* rs1064261								
	AA	916 (82.2)	945 (84.8)	0.153	1.00		1.00	0.134
	AG	194 (17.4)	164 (14.7)		1.22 (0.97–1.53)	0.085	1.22 (0.96–1.55)	0.098
	GG	4 (0.4)	6 (0.5)		0.69 (0.19–2.45)	0.563	0.87 (0.24–3.22)	0.840
	AG/GG	198 (17.8)	170 (15.2)		1.20 (0.96–1.50)	0.109	1.21 (0.96–1.53)	0.108
Combined effect of risk genotypes^c^								
	0	273 (24.6)	322 (29.1)		1.00		1.00	0.107
	1	362 (32.6)	338 (30.6)		**1.27 (1.03**–**1.58)**	**0.029**	**1.34 (1.07**–**1.68)**	**0.012**
	2	177 (16.0)	157 (14.2)		**1.34 (1.03**–**1.75)**	**0.032**	**1.42 (1.07**–**1.87)**	**0.014**
	3	209 (18.9)	200 (18.1)		1.24 (0.97–1.60)	0.089	**1.29 (0.99**–**1.67)**	**0.058**
	4	83(7.48)	83 (7.50)		1.19 (0.84–1.68)	0.322	1.21 (0.85–1.73)	0.293
	5	5 (0.45)	6 (0.54)		0.99 (0.30–3.28)	0.988	1.27 (0.35–4.54)	0.717
					*P* ^trend^ = 0.181		*P* ^trend^ = 0.107	
	0	280 (25.1)	333 (29.8)		1.00		1.00	
	≥1	836 (74.9)	784 (70.2)		**1.27 (1.05**–**1.53)**	**0.013**	**1.33 (1.10**–**1.61)**	**0.004**

^a^For additive genetic models. The results were in bold, if the 95% CI excluded 1 or *P* < 0.05.

^b^Adjusted for age, sex, smoking and drinking status in logistic regress models.

^c^Risk genotypes used for the calculation were *PIK3R1* rs3730089 AG/AA + *PIK3R1* rs3730090 CT/TT + *mTOR* rs2295080 GT/G G + *mTOR* rs1057079 CT/CC + *mTOR* rs1064261 AG/GG.

**Table 3:** Stratification analysis for associations between variant genotypes of *PIK3R1*and ESCC risk

	*PIK3R1* 3730089							*PIK3R1* 3730090						
	(cases/controls)							(cases/controls)						
Variables	GG	AG/AA	Crude OR (95% CI)	*P*	Adjusted OR (95% CI)	*P a*	*P_hom_*	CC	CT/TT	Crude OR (95% CI)	*P*	Adjusted OR*a* (95% CI)	*P a*	*P_hom_*
Age														
≤60	373/365	192/190	0.99 (0.77–1.23)	0.929	0.97 (0.74–1.25)	0.790	0.853	415/420	150/132	1.13 (0.87–1.49)	0.363	1.20 (0.90–1.59)	0.222	0.455
>60	363/364	187/196	0.96 (0.75–1.23)	0.726	1.03 (0.79–1.34)	0.844		422/429	128/131	0.98 (0.74–1.29)	0.873	0.96 (0.72–1.28)	0.770	
Sex														
Females	153/152	65/82	0.79(0.53–1.17)	0.236	0.78 (0.51–1.18)	0.248	0.174	158/181	60/53	1.27 (0.83–1.95)	0.266	1.40 (0.88–2.22)	0.153	0.337
Males	582/576	314/305	1.02 (0.84–1.24)	0.825	1.08 (0.88–1.33)	0.450		679/668	217/213	1.01 (0.81–1.25)	0.948	0.98(0.78–1.23)	0.871	
Smoking status														
Never	286/321	149/182	0.92 (0.70–1.20)	0.538	0.92 (0.70–1.21)	0.542	0.556	318/386	115/115	1.21 (0.90–1.64)	0.203	1.25 (0.92–1.71)	0.157	0.239
Ever	450/408	230/204	1.02 (0.81–1.29)	0.852	1.06 (0.83–1.36)	0.620		519/463	159/148	0.96 (0.74–1.24)	0.745	0.99 (0.76–1.30)	0.957	
Drinking status														
Never	416/494	202/252	0.95 (0.76–1.19)	0.670	0.97 (0.76–1.22)	0.776	0.257	450/565	166/178	1.17 (0.92–1.50)	0.207	1.16 (0.90–1.50)	0.256	0.271
Ever	298/235	199/134	0.97 (0.73–1.28)	0.832	1.09 (0.81–1.47)	0.574		387/284	108/85	0.93 (0.68–1.29)	0.670	0.90 (0.63–1.25)	0.507	
BMI														
<25.0	485/320	235/164	0.95(0.74–1.21)	0.677	0.95(0.74–1.22)	0.696	0.583	541/365	179/119	1.02 (0.78–1.33)	0.903	1.02 (0.78–1.33)	0.897	0.784
≥25.0	251/409	143/222	1.05(0.81–1.37)	0.717	1.03 (0.79–1.35)	0.824		297/484	97/147	1.08 (0.80–1.45)	0.632	1.10 (0.82–1.49)	0.530	

CI, confidence interval; OR, odds ratio.

^a^Obtained in logistic regression models with adjustment for age, sex, smoking status and drinking status.

*P*_hom _derived from the homogeneity test.

The results were in bold, if the 95% CI excluded 1 or *P* < 0.05.

**Table 4:** Stratification analysis for associations between variant genotypes of *mTOR* and ESCC risk

	*mTOR* rs1057079							*mTOR* rs1014261							
	(cases/controls)							(cases/controls)							
Variables	TT	CT/CC	Crude OR (95% CI)	*P*	Adjusted OR (95% CI)	*P a*	*P_hom_*	AA		AG/GG	Crude OR (95% CI)	*P*	Adjusted OR*a* (95% CI)	*P a*	*P_hom_*
Age															
≤60	364/362	201/193	1.04 (0.81–1.32)	0.780	1.05 (0.81–1.37)	0.692	0.550	464/468		101/87	1.16 (0.84–1.58)	0.368	1.20 (0.86–1.67)	0.276	0.727
>60	338/363	212/197	1.15 (0.90–1.47)	0.261	1.15 (0.89–1.48)	0.284		451/477		99/83	1.25 (0.91–1.72)	0.172	1.27 (0.91–1.77)	0.167	
Sex															
Females	142/150	76/84	0.96 (0.65–1.41)	0.818	1.01 (0.67–1.53)	0.956	0.453	179/201		39/33	1.33 (0.80–2.20)	0.273	1.24 (0.71–2.15)	0.451	0.665
Males	560/575	336/306	1.13 (0.93–1.37)	0.225	1.13 (0.93–1.39)	0.226		737/744		159/137	1.17 (0.91–1.50)	0.215	1.17 (0.99–1.52)	0.243	
Smoking status															
Never	267/314	166/188	1.04 (0.80–1.35)	0.780	1.02 (0.77–1.34)	0.897	0.566	352/414		81/87	1.10 (0.79–1.53)	0.594	1.08 (0.77–1.52)	0.678	0.405
Ever	435/411	246/202	1.15 (0.91–1.45)	0.231	1.21 (0.95–1.54)	0.132		564/531		117/83	1.33 (0.98–1.80)	0.069	1.31 (0.95–1.80)	0.104	
Drinking status															
Never	388/480	228/266	1.06 (0.85–1.32)	0.604	1.08 (0.86–1.36)	0.516	0.631	506/636		110/110	1.25 (0.94–1.68)	0.121	1.24 (0.92–1.66)	0.167	0.585
Ever	314/245	184/124	1.16 (0.87–1.54)	0.309	1.16 (0.86–1.57)	0.322		410/309		88/60	1.11 (0.77–1.58)	0.585	1.10 (0.75–1.61)	0.632	
BMI															
<25.0	437/323	283/161	**1.30 (1.02**–**1.65)**	**0.033**	**1.31 (1.03**–**1.67)**	**0.031**	**0.022**	585/415		135/69	**1.39 (1.01**–**1.91)**	**0.042**	**1.39 (1.01**–**1.92)**	**0.043**	0.167
≥25.0	265/402	129/229	0.86 (0.66–1.12)	0.246	0.87 (0.67–1.14)	0.311		331/530		63/101	1.00 (0.71–1.41)	0.994	0.99 (0.70–1.40)	0.962	

CI, confidence interval; OR, odds ratio.

^a^Obtained in logistic regression models with adjustment for age, sex, smoking status and drinking status.

*P*_hom _derived from the homogeneity test.

The results were in bold, if the 95% CI excluded 1 or *P* < 0.05.

**Table 5:** Stratification analysis for associations between variant genotypes of *mTOR* and ESCC risk

	*mTOR* rs2295080							Combined risk genotypes							
	(cases/controls)							(cases/controls)							
Variables	TT	GT/GG	Crude OR (95% CI)	*P*	Adjusted OR (95% CI)	*P a*	*P_hom_*	0	≥1	Crude OR (95% CI)	*P*	Adjusted OR*a* (95% CI)	*P a*		*P_hom_*
Age															
≤60	342/350	223/205	1.12 (0.88–1.42)	0.363	1.14 (0.89–1.48)	0.303	0.092	325/341	240/214	1.18 (0.93–1.49)	0.182	1.20 (0.93–1.55)	0.157		0.674
>60	332/345	218/205	1.11 (0.87–1.41)	0.414	1.12 (0.87–1.44)	0.381		312/350	238/210	1.27 (1.00–1.61)	0.055	**1.28 (1.01**–**1.65)**	**0.049**		
Sex															
Females	136/146	82/88	1.00 (0.68–1.46)	1.000	1.05 (0.70–1.57)	0.813	0.542	134/146	84/88	1.04 (0.71–1.52)	0.837	1.11 (0.74–1.66)	0.631		0.362
Males	538/556	358/325	1.14 (0.94–1.38)	0.174	1.14 (0.94–1.38)	0.181		504/546	392/335	**1.27 (1.05**–**1.53)**	**0.014**	**1.28 (1.05**–**1.56)**	**0.014**		
Smoking status															
Never	249/303	184/198	1.13 (0.87–1.47)	0.357	1.12 (0.85–1.47)	0.419	0.972	237/299	198/204	1.22 (0.95–1.59)	0.126	1.21 (0.92–1.58)	0.166		0.937
Ever	425/399	255/213	1.12 (0.90–1.41)	0.315	1.18 (0.93–1.51)	0.172		401/393	280/221	1.24 (0.99–1.55)	0.059	**1.31 (1.03**–**1.67)**	**0.028**		
Drinking status															
Never	376/467	240/277	1.08 (0.86–1.34)	0.513	1.11 (0.88–1.39)	0.388	0.640	357/460	261/288	1.17 (0.94–1.45)	0.162	1.20 (0.95–1.50)	0.119		0.527
Ever	298/235	199/134	1.17 (0.89–1.55)	0.265	1.20 (0.90–1.62)	0.216		281/232	217/137	1.31 (0.99–1.72)	0.056	**1.34 (1.00**–**1.79)**	**0.051**		
BMI															
<25.0	420/315	300/169	**1.34(1.06**–**1.70)**	**0.016**	**1.36 (1.07**–**1.73)**	**0.014**	0.016	392/312	328/172	**1.51 (1.19**–**1.91)**	**0.001**	**1.52 (1.20**–**1.94)**	**0.001**		**0.006**
≥25.0	256/386	138/245	0.87(0.67–1.13)	0.285	0.88 (0.67–1.14)	0.323		244/380	150/251	0.92 (0.71–1.29)	0.544	0.95 (0.73–1.24)	0.711		

CI, confidence interval; OR, odds ratio.

^a^Obtained in logistic regression models with adjustment for age, sex, smoking status and drinking status.

*P*_hom _derived from the homogeneity test.

The results were in bold, if the 95% CI excluded 1 or *P* < 0.05.

**Table 6:** MDR analysis for the prediction of ESCC risk with and without *PIK3R1 & mTOR* genotypes

Best interaction models	Cross-validation	Average prediction error	*P a*
1	100/100	0.394	< 0.0001
1, 2	100/100	0.394	< 0.0001
1, 2, 3	98/100	0.386	< 0.0001
1, 2, 3, 4	93/100	0.379	< 0.0001
1, 2, 3, 4, 5	100/100	0.370	< 0.0001
1, 2, 3, 4, 5, 6	100/100	0.367	< 0.0001
1, 2, 3, 4, 5, 6, 7	92/100	0.358	< 0.0001
1, 2, 3, 4, 5, 6, 7, 8	94/100	0.350	< 0.0001
1, 2, 3, 4, 5, 6, 7, 8, 9	100/100	0.341	< 0.0001
**1, 2, 3, 4, 5, 6, 7, 8, 9, 10**	100/100	0.340	< 0.0001

MDR, multifactor dimensionality reduction.

^a^*P* value for 1000-fold permutation test.

The best model with maximum cross-validation consistency and minimum prediction error rate was in bold.

Labels: 1, BMI; 2, smoking status; 3, sex; 4, age; 5, drink status; 6, *PIK3R1* rs3730089; 7, *mTOR* rs2295080; 8, *PIK3R1* rs3730090; 9, *mTOR* rs1057079; 10, *mTOR* rs1064061.

**Table 7:** Haplotype analysis for genotypes of *mTOR* and ESCC risk

	Haplotype frequencies							
	Cases (N = 2232)		Controls (N = 2234)					
Haplotypes a	n	%	n	%	Crude OR (95% CI)	*P*	Adjusted OR (95% CI)	*P* a
T-T-A	1700	76.23	1746	78.72	1.00	0.009	1.00	
T-C-A	23	1.03	9	0.41	2.63 (1.21–5.69)	0.015	2.96 (1.32–6.67	**0.023**
T-C-G	19	0.85	6	0.27	3.25 (1.30–8.16)	0.012	2.97 (1.16–7.60)	**0.024**
G-T-A	40	1.79	18	0.81	2.28 (1.30–4.00)	0.004	2.41 (1.34–4.37)	**0.003**
G-T-G	33	1.48	27	1.22	1.26 (0.75–2.10)	0.385	1.38 (0.81–2.35)	0.234
T-C-G	265	11.88	271	12.22	1.01 (0.84–1.21)	0.963	1.03 (0.85–1.24)	0.795
G-T-A	150	6.73	141	6.36	1.09 (0.86–1.39)	0.468	1.12 (0.87–1.43)	0.380

^a^Obtained in logistic regression models with adjustment for age, sex, smoking status and drinking status.

**Table 8:** Gene-gene and gene-environment interactions (logistic regression)

*P* value								
	rs3730089	rs3730090	rs2295080	rs1057079	rs1064261	Drinking	Smoking	BMI
rs3730089								
rs3730090	0.8746							
rs2295080	0.3692	0.1886						
rs1057079	0.6180	0.1129	**<0.001**					
rs1064261	0.7348	0.5803	**0.0246**	0.7329				
Drinking	0.9182	0.2707	0.6398	0.6315	0.5853			
Smoking	0.5558	0.2390	0.9725	0.5663	0.4052	**0.0016**		
BMI	0.8435	0.1294	**0.0046**	**0.001**	0.0797	0.9281	0.3237	
